# Roles of Endovascular Calyx Related Enzymes in Endothelial Dysfunction and Diabetic Vascular Complications

**DOI:** 10.3389/fphar.2020.590614

**Published:** 2020-11-30

**Authors:** Zhi Li, Ning Wu, Jing Wang, Quanbin Zhang

**Affiliations:** ^1^ Key Laboratory of Experimental Marine Biology, Center for Ocean Mega-Science, Institute of Oceanology, Chinese Academy of Sciences, Qingdao, China; ^2^ Lab for Marine Biology and Biotechnology, Qingdao National Lab for Marine Science and Technology, Qingdao, China; ^3^ University of Chinese Academy of Sciences, Beijing, China; ^4^ Laboratory for Marine Drugs and Bioproducts, Pilot National Laboratory for Marine Science and Technology, Qingdao, China

**Keywords:** glycocalyx, vascular complications, diabetes, endothelial dysfunction, enzyme

## Abstract

In recent years, the number of diabetic patients has rapidly increased. Diabetic vascular complications seriously affect people’s quality of life. Studies found that endothelial dysfunction precedes the vascular complications of diabetes. Endothelial dysfunction is related to glycocalyx degradation on the surface of blood vessels. Heparanase (HPSE), matrix metalloproteinase (MMP), hyaluronidase (HYAL), hyaluronic acid synthase (HAS), and neuraminidase (NEU) are related to glycocalyx degradation. Therefore, we reviewed the relationship between endothelial dysfunction and the vascular complications of diabetes from the perspective of enzymes.

## Highlights

The glycocalyx attaches to the intima of blood vessels, senses shear stress, regulates signaling factors, and protects endothelial cells.
High sugar and an inflammatory environment cause glycocalyx-degrading enzymes to be upregulated, triggering glycocalyx degradation.
Degradation of the glycocalyx produces heparan sulfate, syndecan fragments which can trigger inflammatory pathway NF-КB, which in turn leads to many vascular complications.
In vascular complications, glycocalyx-degrading-enzyme expression is upregulated, which further promotes glycocalyx degradation.

## Introduction

The intimal vascular surface, including the endothelial glycocalyx and other soluble components, is attached to vascular endothelial cells, and is between the blood and blood vessels (Zhang J. et al., 2019). Among them, other soluble components include superoxide dismutase, hyaluronic acid, and albumin adhered to glycogen in plasma (Reitsma et al., 2007; Sieve et al., 2018a).

The endothelial glycocalyx is a porous, hairlike, regularly organized layer. The glycocalyx is negatively charged and sheds off to equilibrium under physiological conditions (Lebel, 1991; Zeng, 2017). The endothelial glycocalyx is composed of proteoglycans (skeleton proteins that maintain the relationship between glycosaminoglycan and endothelial cells), glycoproteins (endothelial cell-adhesion molecules, vascular cell-adhesion molecules, and platelets), and glycolipids (Dogne et al., 2018). Proteoglycans consist of glycosaminoglycans (heparan sulfate, chondroitin sulfate, hyaluronic acid) and protein ligands (syndecans) (Goligorsky, 2017; Dogne et al., 2018). Research found that the main component of proteoglycans in glycocalyx is heparin sulfate, while chondroitin sulfate and hyaluronic acid account for a relatively small amount (Kalagara et al., 2018), and the ratio of heparan sulfate and chondroitin sulfate in vascular endothelium is 4:1 (Sladden et al., 2019). Nonsulfated glycosaminoglycan hyaluronic acid is noncovalently linked to glycoprotein CD44, and can be exchanged with the bloodstream, while heparin and chondroitin sulfate are covalently linked to the proteoglycan core protein (Yang X. et al., 2018). Studies found that glycocalyx plays an important role in protecting the integrity and permeability of vascular endothelial cells; in addition, it can regulate blood flow, reduce inflammation, and regulate signal transduction (Mulivor and Lipowsky, 2002; Curry and Adamson, 2012; Salmon et al., 2012; Sieve et al., 2018a; Dogne et al., 2018).

Diabetes is chronic vascular inflammation. Long-term infiltrating blood vessels in a high-glucose environment causes endothelial dysfunction, glycocalyx destruction, and vascular complications. Some reports found that diabetic vascular complications (atherosclerosis, thrombosis, diabetic foot, diabetic retinopathy, and diabetic nephropathy)sepsis, (surgical) ischemia/reperfusion injury, trauma, and acute lung injury could all cause glycocalyx degradation (Cancel et al., 2016; Wang et al., 2016; Sieve et al., 2018a). Likewise, glycocalyx degradation can cause vascular complications (Raffetto and Mannello, 2014; Cochain and Zernecke, 2017; Shirazi et al., 2017; Sieve et al., 2018a). Furthermore, Futhermoer, these diseases involve inflammatory stimulation (NF-КB pathway), oxidative stress (endothelial nitric oxide synthase (eNOS) uncoupling), and shear stress (AMP-activated protein kinase (AMPK) pathway) (Ramnath et al., 2014; Sieve et al., 2018a). The harmful effects of diabetes or acute hyperglycemia on the human and mice endothelial cell glycocalyx were confirmed in multiple studies (Nieuwdorp et al., 2006a; Nieuwdorp et al., 2006b; Dogne et al., 2016). The glycocalyx coverage area of db/db mice decreases in the early stages of diabetes, which triggers a change in permeability, while the length of glycocalyx decreases in the late stages of diabetes (Targosz-Korecka et al., 2017). However, Wadowski et al. (2019) stated that changes in the glycocalyx are related to age. In addition, the degradation of glycocalyx causes active substances such as xanthine oxidase, lipoprotein lipase, tissue factor pathway inhibitor, fibroblast growth factors, vascular endothelial growth factor, which are attached to the surface of the glycocalyx, to diffuse into the blood, changing from local to systemic activity (Lupu et al., 1999; Battelli et al., 2014; Becker et al., 2015). Previous research found that hyaluronidase (Dogne et al., 2016), heparanases (Arfian et al., 2019), matrix metalloproteinases (Ramnath et al., 2014), neuraminidase (Sieve et al., 2018a), and hyaluronic acid synthetase (Zhang et al., 2018) are closely related to the degradation of glycocalyx. When these enzymes are missing or their expression is inhibited, glycocalyx degradation is reduced. Therefore, we summarize the degradation of glycocalyx from the perspective of enzymes, and review the relationship between enzymes and glycocalyx damage, and the mechanisms involved in it, providing a new direction for understanding complications of diabetes.

## Hyaluronidase

Nonprotein-bound hyaluronic acid is a linear, nonsulfate, negatively charged glycosaminoglycan that consists of glucuronic acid and n-acetylglucosamine repeating units. In the glycocalyx, hyaluronic acid binds to the CD44 receptor, but it is not covalently linked and can freely exchange with the bloodstream (Sladden et al., 2019). Studies showed that the human body contains six types of hyaluronidase, which completes the degradation of hyaluronic acid. However, HYAL1 and HYAL2 are the main hyaluronidases in mammalian tissue. They cooperate with each other to complete the degradation of hyaluronic acid (Cai et al., 2019). Among them, hyaluronidase 2 is a glycosylphosphatidylinositol anchor enzyme attached to the outer surface of the plasma membranes (Kong et al., 2016), and it is responsible for degrading extracellular high-molecular-weight hyaluronic acid into a medium fragment of about 20 kDa. Then, intermediate hyaluronic acid fragments are endocytosed into cells by endocytosis vesicles, and degraded into small fragments by HYAL1 (Patel et al., 2013). Studies showed that HYAL1 is the only hyaluronidase present in the plasma and urine of mammals, and it is also high in major organs such as the liver, kidneys, spleen, and heart (Cai et al., 2019). HYAL1 is endocytosed into lysosomes and is active when pH < 4 (Puissant et al., 2014; Dogne et al., 2016), while the optimal pH of HYAL2 is 6.0–7.0 < 4 (Puissant et al., 2014; Dogne et al., 2016). Furthermore, a previous paper pointed out that human platelets contain hyaluronidase 2, while other cells contain HYAL1 and HYAL2 (Wang G.-H. et al., 2019).

In hyperglycemic and/or inflammatory environments, hyaluronidase expression is upregulated, cutting the glycocalyx and producing hyaluronic acid fragments. Previous studies found that hyaluronic acid and hyaluronidase activity are increased in the serum of diabetics patients (Nieuwdorp et al., 2006a) and mice (Ikegami-Kawai et al., 2003; Leskova et al., 2019). Increased plasma hyaluronic acid and hyaluronidase activity were detected in both human Type 1 (Nieuwdorp et al., 2006a) and Type 2 (Broekhuizen et al., 2010) diabetes. Hyaluronidase 1 inhibitors may play a role in diabetic endothelial dysfunction in hyaluronidase-deficient mice, and supplementation with hyaluronic acid analogs can reduce glycocalyx loss (Dogne et al., 2016). Reducing glycocalyx degradation could also be observed in sulodexide-treated mice, in which hyaluronidase activity was decreased in plasma (Henry and Duling, 1999; Broekhuizen et al., 2010). Monzon et al. (2008) found that TNF-α and IL-β coordinately upregulate HYAL 1, 2, 3, and TNF-α induces HYAL1 mRNA expression. Lokeshwar et al. (2008) pointed out that NF-КB is located upstream of HYAL, which indicates that the inflammatory response can upregulate the expression of HYAL. Therefore, reducing the expression of hyaluronidase can reduce glycocalyx degradation.

Hyaluronidase cleaves the glycocalyx to produce low-molecular-weight hyaluronic acid fragments; the properties of high- and low-molecular-weight hyaluronic acid are very different. The former enhances the barrier function of endothelial cells, while the latter destroys endothelial cells, induces toll-like receptors 2 and 4, and then induces cell inflammation (Zhang et al., 2018). Low-molecular-weight hyaluronic acid fragments may in turn stimulate reactive-oxygen-species production in a size-dependent manner by phagocytes. Furthermore, hyaluronic acid fragments could induce the expression of vascular cell-adhesion molecule - 1 and intercellular-adhesion molecule - 1, caused an increase in macrophages, and further cause inflammation and damage to endothelial cells (Sieve et al., 2018a; Ali et al., 2019; Zhang H.-M. et al., 2019). Targosz-Korecka et al. (2020) showed that the recovery of the endothelial glycocalyx is related to the reduced expression of e-selectin and intercellular-adhesion molecule 1. Many researchers intercellular adhesion moleculediscovered that shear stress induces an increase in the amount of hyaluronidase enzyme (Sieve et al., 2018a; Ali et al., 2019; Zhang H.-M. et al., 2019). Kong et al. (2016) found that HYAL2 is related to eNOS-Ser-633 dephosphorylation. They found that low shear stress induced the upregulation of HYAL2 expression, which in turn led to eNOS-Ser-633 dephosphorylation, and then to the downregulation of NO expression. The alteration of eNOS-Ser-633 dephosphorylation caused by low shear stress was eliminated in human umbilical vein endothelial cells (HUVECs) transfected with HYAL2 siRNA (Kong et al., 2016). Yang H. et al. (2018) discovered that shear stress can reduce AMPK *α*-Thr-172 phosphorylation (this process is regulated by the ERK1/2 or CD44 pathway), which leads to AMPK inactivation. Then, it activates the Na^+^-H^+^ exchanger 1 (NHE1) pathway (NHE is an ion protein channel that can cause H^+^ efflux; extracellular pH is downregulated), causing an increase expression in HYAL2, which degrades hyaluronic acid and causes endothelial-glycocalyx damage. Yang H. et al. (2018) pointed out that low shear stress decreases AMPK Thr172 phosphorylation levels and increases p47^phox^ activation in HUVECs to upstream HYAL2, while the siRNA knockout of p47^phox^ reduces hyaluronic acid activation. A previous paper deemed that, with the increase in shear force, the content of hyaluronidases, heparanases, and chondroitinases in the endothelial-cell glycocalyx gradually increases, resulting in higher glycocalyx thickness at high-shear-force sites (Yang H. et al., 2018). However, the thickness of the glycocalyx of endothelial cells in the low-shear-force region is small, which may lead to weakened vascular protection, and increased cell adhesion and lipid deposition, making this site more prone to atherosclerosis (Zeng, 2017).

In conclusion, hyperglycemia can induce the expression of hyaluronidases 1 and 2 in humans and mice, and then lead to glycocalyx degradation and endothelial-cell dysfunction. In diabetic patients and mice, low shear stress can cause the upregulation of hyaluronidase 2 expression, and low-shear-stress-mediated changes in the amount of enzymes are related to phosphorylated proteins, which suggests that we can study the relationship between hyperglycemia and the amount of hyaluronidase from the perspective of transcription. The cardiovascular complications of diabetes, such as atherosclerosis, can cause changes in shear stress, while low shear stress can further cause endothelial-glycocalyx destruction and vascular disease.

## Heparanases

Heparanases are the only enzyme that degrades heparan sulfate-endo-β-D glucuronide in mammals. Heparanase gene knockout mice also confirmed that only one gene encodes a heparanase with endoglycosidase activity (Zcharia et al., 2009; Gil et al., 2012; Zengbo, 2014; Rabelink et al., 2017). Heparanases interactors are formed by a network of 300 proteins. HPSE upregulates many adverse diseases, including diabetes, sepsis, and cancer (Levy-Adam et al., 2003; Zengbo, 2014). HPSE is expressed as a 65 kD inactive precursor and processed into a 50 kD active isoform by the cleavage of cathepsin L 6 (Garsen et al., 2016; van den Berg, 2019). After heparanase is activated, its active form is released from the late inclusion body or lysosome to the outside of the cell. This process is activated by the extracellular pathway activating protein kinase Aand protein kinase C signaling pathways (Wan et al., 2008). In addition, heparanase can be regulated by regulating gene expression *in vivo*. The factors that regulate gene expression may include inflammatory cytokines and early-growth-response transcription factors (Baker et al., 2009; Lerolle et al., 2010). Hypoxia can also cause increased heparanase expression (Simeonovic et al., 2013).

Heparanases are highly related to diabetes and inflammation; under high glucose conditions, the expression of heparinase can be upregulated in adipose tissue (Arfian et al., 2019), endothelial cells (Zhu et al., 2019), and podocytes (Yoshibayashi et al., 2020), which leads to a decrease in the expression of syndecan and a glycocalyx disorder. Experiments showed that vitamin D (Garsen et al., 2015), atlasacetam (Boels et al., 2016), and hypericin (An et al., 2017) can reduce damage to the endothelial glycocalyx by inhibiting heparanases. Diabetes can cause increased expression of TNF-α, IL-1β, and NF-КB p65 phosphorylation (Niu et al., 2019). When inflammation occurs, proinflammatory factors (TNF-α) activate vascular endothelial cells, P- and E-selectin expression increases, leukocytes begin to roll and attach to endothelial cells, and chemokines on endothelial cells start to activate leukocytes (Singh et al., 2011; Pahwa et al., 2016). Leukocytes would be activated through adhesion molecules on endothelial cells, such as vascular cell-adhesion molecule 1 and intercellular-adhesion molecule 1, which stably adhere to the vascular endothelium (Wang T.-T. et al., 2019; El Masri et al., 2020). Once white blood cells stay on the surface of the endothelial cells, they begin to penetrate the endothelial cell layer and enter the interstitial tissue, causing inflammatory damage to the organs. Therefore, vascular endothelial cells play an important role in the occurrence of inflammatory damage to the kidneys (Zhang et al., 2018). Zengbo (2014) pointed out that heparanase expression in the kidneys of sepsis mice increased significantly earlier than it did in the adhesion molecules. Heparanase is an important factor in the pathogenesis of acute kidney injury in sepsis (Kinsey and Okusa, 2012). The mechanism may be that, after sepsis, TNF-α increases the activation of heparanase in renal interstitial microvascular endothelial cells, and glycocalyx degradation causes interstitial leukocyte infiltration, and glomerular filtration barrier disruption can then cause proteinuria. Heparan sulfate analogs can also improve renal-function injury in patients with sepsis, and improve the survival rate of patients with sepsis by inhibiting heparanase activity (Zengbo, 2014). Therefore, heparinase inhibitors and heparin analogs can reduce glycocalyx loss.

The abnormal expression of heparanase can cause atherosclerosis. In endothelial cells and foam macrophages of atherosclerotic lesions, inflammatory stimulation leads to the upregulation of angiopoietin 2, which leads to increased expression of heparanases, leading to the degradation of heparan sulfate on the arterial wall (Rao et al., 2011; Sieve et al., 2018a). Then, the released HS can lead to the activation of leukocytes and platelets, and increase the expression of intercellular-adhesion molecule 1 and vascular cell-adhesion molecule 1, which leads to the adhesion of white blood cells to endothelial cells, causing low-molecular-weight infiltration, eventually causing high blood pressure (Chappell et al., 2010; McDonald et al., 2016; Sieve et al., 2018a). In addition, glycocalyx shedding produces low-molecular-weight hyaluronic acid and heparan sulfate fragments that act as ligands for CD44, toll-like receptors 2 and 4 (Goldberg et al., 2014; Pahwa et al., 2016; Rabelink et al., 2017), and advanced glycation end-productreceptors. These lead to the expression of cytokines in monocytes and macrophages, which promotes the upregulation of NF-КB expression, leading to an increase in TNF-α and IL-6 expression (Yamawaki et al., 2009; Docampo et al., 2017). This causes the upregulation of heparanases again, causing the whole glycocalyx to fall off (Uchimido et al., 2019). However, the increase in TNF-α and IL-6 causes the expression of MMP and the production of reactive oxygen species (Ramnath et al., 2014), activates mast cells, promotes the degranulation of mast cells, and further releases cytokines, histamine, protease, heparanases, and other components of glycocalyx degradation to destroy glycocalyx endothelial cells (Gil et al., 2012). This is a vicious cycle that further causes endothelial surface-layer layer degradation (Sieve et al., 2018a). Some researchers also pointed out that heparin and heparin analogs could reduce plasma syndecan 1 and heparan sulfate levels in sepsis, and reduce glycocalyx damage (Gil et al., 2012).

There are some other pathways related to heparanases. One case involves sirtuin 1, which is related to transcription. Sirtuin 1 deacetylates p65, which reduces the transcriptional activity of NF-КB to heparanases. Sirtuin 1 deficiency activates NF-КB, which in turn promotes the transcriptional expression of heparanases (Zhang et al., 2018). In addition, Manchanda et al. (2018) discovered that myeloperoxidase can cause neutrophil-dependent syndecan 1 shedding and endothelial-glycocalyx structural collapse through ionic interactions with heparan sulfate side chains. Gil et al. (2012) pointed out that, in a diabetic nephropathy model, the early-growth response of transcription factor 1 activates the heparanase promoter. However, specific heparanase inhibitor SST0001 can significantly reduce the degree of proteinuria and kidney damage in diabetic-nephropathy mice. Furthermore, extracellular heparanases are involved in the inflammatory response of acute kidney injury, but may also be involved in the long-term adverse consequences of acute kidney injury, such as fibrosis and the final development of chronic kidney disease, suggesting that heparanases as a potential therapeutic target for acute kidney injury deserve further exploration (Rabelink et al., 2017).

Diabetes is a chronic vascular inflammation, and blood vessels that are infiltrated in a hyperglycemic environment for a long time become diseased. High glucose induces inflammatory stimuli (El Masri et al., 2020), leading to increased expression of inflammatory factors IL-6 and TNF-α. The increase in these factors is related to AMPK, NF-КB, and STAT3 (reactive oxygen species) signaling pathways, and TNF-α modifies the related mitochondrial respiratory chain redox response role (Szot et al., 2019). Intracellular heparanases have many important biological functions, including regulating autophagy, and cell communication and survival. In contrast, extracellular heparanases are directly related to inflammation, vascular instability, and fibrosis, and are a key factor in the occurrence of proteinuria and renal damage in patients with diabetic nephropathy and inflammatory glomerulonephritis (Rabelink et al., 2017). Upregulation of the expression of heparanases induced by inflammatory factors causes glycocalyx degradation. While triggering a series of vascular complications, the hyaluronic acid fragment produced by glycocalyx degradation and heparanase production form a vicious cycle. Therefore, it is of great significance to study the mechanism of glycocalyx degradation and heparanase expression changes caused by hyperglycemia.

## Matrix Metalloproteinases

Matrix metalloproteinases are a class of zinc-dependent endopeptidases that degrade collagen, gelatin, and elastin in the extracellular matrix, thereby promoting vascular remodeling (Castro et al., 2008). Matrix metalloproteinases not only degrade extracellular-matrix components leading to vascular-wall instability, but also damage the monolayer glycocalyx integrity of endothelial cells (Chen et al., 2017; Sieve et al., 2018a). Matrix metalloproteinases are generally expressed in inflammatory cells, but can also be expressed in endothelial cells and vascular smooth muscle cells after being stimulated by factors secreted by macrophages. Matrix metalloproteinases can cleave proteoglycan core proteins (such as syndecan). Usually, MMP-9 causes the degradation of syndecan-1, while MMP-2 causes the cleavage of syndecan-4 (Yuelong et al., 2014). This allows for syndecan and heparan sulfate to be released into the bloodstream, causing a decrease in heparan sulfate and syndecan in the glycocalyx (Ramnath et al., 2014). On the one hand, the reduction of negatively charged heparan sulfate in the glycocalyx results in the loss of heparan sulfate-bound extracellular SOD (Mulivor and Lipowsky, 2009; Ramnath et al., 2014); on the other hand, heparan sulfate fragments and syndecan can cause thrombosis and endothelial inflammation (Hsia et al., 2017; Sieve et al., 2018a).

Diabetes and some inflammation can cause reactive oxygen species. Reactive oxygen species can activate matrix metalloproteinases and inactivate the tissue inhibitors of metalloproteinases (TIMPs). In addition, reactive oxygen species can change the phenotype of mesangial cells and impair podocyte integrity which related to diabetes nephropathy (Nagasu et al., 2016). Reactive oxygen species activate MMP precursors such as pro-MMP-2 to activate MMPs, which in turn causes vascular remodeling and inflammation (Cai et al., 2019). This can cause macrovascular disease in diabetic patients. Furthermore, studies found that, in vascular smooth muscle cells, reactive oxygen species can induce MMP-2 mRNA expression (Grote et al., 2003), which highlights the mechanism that reactive oxygen species could regulate matrix metalloproteinase activity on the transcriptional level (Sieve et al., 2018a). Diabetes-induced reactive oxygen species could also cause nitrosation and oxidative stress, leading to endothelial cell dysfunction (Zeng, 2017). These are closely related to diabetic vascular complications.

The abnormal expression of matrix metalloproteinases triggers cardiovascular disease, which is a diabetic macrovascular disease. In diabetic macrovascular disease, low shear stress induces the initial damage, and high shear stress promotes the formation of vulnerable plaques. At the site of vascular disease, continuous exposure of endothelial cells to high shear stress causes the abnormal production of NO, which may be related to the degradation of the glycocalyx and extracellular matrix by matrix metalloproteinases, and may also be related to inflammation (Zeng, 2017). This process is also related to reactive oxygen. On the one hand, mitochondrial reactive oxygen species can lead to eNOS uncoupling, which in turn reduces NO bioavailability, increases the formation of peroxynitrite, causes vascular relaxation and cause cardiovascular disease (Cheang et al., 2014). Hyperglycemia can also cause eNOS uncoupling (Yang et al., 2019). NO can activate plasminogen activators and matrix metalloproteinases, inhibit interstitial collagen synthesis, and downregulate TGF-β and plasminogen activator inhibitor 1 (Goligorsky, 2017). On the other hand, reactive oxygen species cause the angiotensin type 2 receptor pathway, resulting in the degradation of heparan sulfate, leading to the loss of extracellular SOD bound by heparan sulfate, further causing increased oxidative stress (Ali et al., 2019). This forms a vicious circle.

Retinopathy in diabetic patients involves two pathways: Sirtuin1/FOXO3 and AMPK phosphorylation. On the one hand, hyperglycemia causes defects in sirtuin 1 activity and reduced p65 activation in the retina, which leads to upregulation of the MMPs pathway, causing mitochondrial damage and apoptosis, thereby promoting apoptosis. Resveratrol can inhibit p65 acetylation, activate sirtuin 1, inhibit the MMP-9 signaling pathway, and reduce retinopathy (Petrovic, 2014). On the other hand, diabetes causes AMPK dephosphorylation, which downregulates the AMPK signaling pathway. This then leads to retinal inflammation (Chen et al., 2019). In addition, studies showed that metformin can upregulate the AMPK pathway in diabetic mice, increase NO utilization, and reduce oxidative stress in the endoplasmic reticulum (Cheang et al., 2014). Therefore, looking for factors from the upstream and downstream of MMP to indirectly inhibit MMP expression is also a good direction.

Degradation of the glycocalyx involved in the matrix metalloproteinase pathway is related to the NF-КB-induced inflammation pathway. Diabetes triggers the release of inflammatory factors. Previous research indicated that, on the surface of the glomerular vascular endothelium (Yuelong et al., 2014), proinflammatory factors such as TNF-α activate mast cells and cause them to release enzymes (hyaluronidase, heparinase, MMP-9/2) (Ramnath et al., 2019; Reine et al., 2019). Then, MMP-9 can destroy the glycocalyx, and release heparan sulfate and syndecan into the blood (Ramnath et al., 2014). This causes endothelial inflammation, vascular endothelial damage, diabetic nephropathy, and proteinuria in the kidney. The administration of matrix metalloproteinase inhibitors can attenuate proteinuria. MMP inhibitor treatment also significantly increased the glycocalyx depth of podocytes, but had no significant effect on glomerular-basement-membrane thickness, podocyte foot processes, and slit-diaphragm width (Ramnath et al., 2019). On the one hand, heparan sulfate fragments bind to CD44, and toll-like receptors 2 and 4 (Pahwa et al., 2016). Toll-like receptors activate the NF-КB pathway, which triggers a vicious cycle of increased TNF and IL-6, and glycocalyx degradation. On the other hand, heparan sulfate fragments bind to the surface of macrophage CD44 and toll-like receptors 2 and 4, activate monocytes and macrophages, promote cell adhesion, and cause hypotonicity (Sieve et al., 2018a).

In a word, MMPs are closely related to the degradation of the glycocalyx induced by inflammatory factors, while diabetes is a chronic inflammatory disease that easily leads to the expression of inflammatory factors. Heparan sulfate and syndecan fragments produced by glycocalyx degradation on the one hand induce a vicious cycle of the NF-КB signaling pathway, and on the other hand cause cell adhesion and hypotonicity. In addition, MMPs are involved in mitochondrial damage caused by reactive oxygen species (ROS), and the AMPK and sirtuin 1 signaling pathways in retinopathy. Therefore, the study of MMPs is of great significance for the study of vascular complications of diabetes.

## Neuraminidase

Sialidases, also known as neuraminidases (NEUs), are a family of enzymes responsible for the regulation of sialic acid expression on the cell surface by removing sialic acid from endogenous glycoconjugates (Xiao et al., 2016). In addition, terminal sialic acid promotes the integrity of the endothelial barrier (Cioffi et al., 2012). The most famous enzyme in this family is influenza neuraminidase, which was first discovered in the 1950s (Parker and Kohler, 2010). Neuraminidases are a large family found in many organisms, including viruses, bacteria, fungi, protozoa, birds, and mammals (Pshezhetsky and Ashmarina, 2013). Neuraminidase is found in many mammalian organs (Pshezhetsky and Ashmarina, 2013; Sieve et al., 2018a). NEU1 is highest expressed in the kidneys, pancreas, skeletal muscle, liver, lungs, placenta, and brain; NEU2 is mainly found in muscle tissue; NEU3 is highest expressed in adrenal glands, skeletal muscle, heart, testes, and thymus; NEU4 is highest expressed in the brain, skeletal muscle, heart, placenta, and liver (Pshezhetsky and Ashmarina, 2013). In cells, NEU1 is localized in lysosomes and plasma membranes to participate in exocytosis, immune response, phagocytosis, and elastic fiber assembly; NEU2 is a soluble protein present in the cytoplasm and plasma membrane, and is involved in the differentiation of myoblasts and neurons; NEU3 is a complete membrane protein localized in the small concave microregions of the plasma membrane, and endolysosomal and lysosomal membranes; NEU4 is localized in lysosomes, mitochondria, or the endoplasmic reticulum. NEU3 and NEU4 are involved in neuronal differentiation, apoptosis, and adhesion (Murayama et al., 1988; Pshezhetsky and Ashmarina, 2013).

The enzymatic hydrolysis of specific substrates in endothelial cells by neuraminidase, heparinase, or hyaluronidase reduces flow-induced nitric oxide (NO) production, which is related to the important role of sialic acid, hyaluronic acid, and heparan sulfate-containing glycosaminoglycans chains in signal transmission (Dogne et al., 2018). Sialidases play a vital role in the interaction and communication between cells (Glanz et al., 2019b). Sialic acid and sulfate are key features of many known glycan recognition motifs (Pshezhetsky and Ashmarina, 2013). The extracellular endothelial glycocalyx can be modified by sulfate, and sulfate is attached to it. The sulfated mode direct receptors bind many key growth factors, including WNT, vascular endothelial, fibroblast, hepatocyte, and heparin-binding epidermal growth factors (Parker and Kohler, 2010). The surface of endothelial cells is highly sialylated, and changes in the state of sialylation, affecting angiogenesis. Human NEU1 is the most abundant sialidase, found in the matrix gel system to inhibit angiogenesis (Lee et al., 2014). It is also the first sialidase to be described as an angiogenesis regulator (Glanz et al., 2019a). Nonstructural protein 1 (Puerta-Guardo et al., 2019) induces sialidase expression, causing sialic acid shedding and endothelial-glycocalyx degradation. Non-structural protein 1 also activates cathepsin L in endothelial cells, a lysosomal cysteine protease that activates heparinase through digestion (Puerta-Guardo et al., 2016). Enzymes that remove or modify these groups can have significant impact on recognition and subsequent signaling events (Parker and Kohler, 2010). Sialic acid prevents the recognition of sugars (such as galactose) and binding proteins (Schauer, 2009). Betteridge et al. (2017) indicated that sialic acid may directly regulate the permeability of the endothelial surface layer, mainly through steric hindrance and/or by inducing secondary changes in this layer, such as the interruption of albumin and glycocalyx binding after dissolution. Therefore, the protective effect of protein-bound sphingosine-1-phosphate on glycogen shedding may be weakened (Betteridge et al., 2017; Sieve et al., 2018a). They also form ligands for selectin, sialic acid-binding immunoglobulin-type lectin (Siglecs), and factor H. Sialic acid is involved in the regulation of complement activation, leukocyte trafficking, and immune cell activation. In addition, the release of sialic acid on the cell surface to regulate glycocalyx, or the release of sialic acid by lysosomes after endocytosis to recover monosaccharides, is mediated by sialidase (Sieve et al., 2018a).

Reconstruction of the extracellular matrix in atherosclerosis is a key step in disease progression. The elastin-receptor complex contains elastin-binding proteins, cathepsin A, and sialidase 1, which mediate endogenous elastin-derived peptides to participate in the chemotactic response of immune cells. Elastin-induced atherosclerosis depends on sialidase activity and the Cath a–neu1 complex. Therefore, elastin can be used as an enhancer of atherosclerosis (Glanz et al., 2019a). The elastin-receptor complex is also essential for the ability of fibroblasts to respond to elastin degradation. The combination of elastin peptides and the elastin-receptor complex activates the intracellular signaling cascade, including the activation of extracellular regulated protein kinases (ERK) 1/2 and the production of pro-MMP-1 (Parker and Kohler, 2010). Sialic acid content in the endothelial glycocalyx plays an important role in the development of atherosclerosis, and the regulation of leukocyte and platelet adhesion, mechanical transduction, and endothelial cell absorption of low-density lipoprotein (Orekhov et al., 2014). Modified low-density lipoprotein also has proinflammatory properties, and is prone to aggregate and form complexes that promote atherosclerosis. Desialylated low-density lipoprotein stays longer in the subendothelial space than unmodified low-density lipoprotein does, which helps in the formation of atherosclerotic plaques (Ivanova et al., 2017). In addition, obesity and Type 2 diabetes are associated with increased serum neuraminidase, an enzyme that increases the output of TGF-β cells (Foote et al., 2016). In addition, NEU1 is a positive enhancer of inflammation, and IL-1 and lipopolysaccharides can induce the expression of NEU1 in monocytes (Sieve et al., 2018b).

Sialic acid is an important signal molecule located on the surface of the cell glycocalyx, which is of great significance for signal recognition and the combination of sugar and protein, while sialidase can cause sialic acid molecules on the glycocalyx surface to fall off. On the one hand, the surface structure of glycocalyx changes, causing obstacles to sialic acid-related signal recognition and glycoprotein binding; on the other hand, the glycocalyx layer is destroyed, causing osmotic disorders. Therefore, the study of sialidase is of great significance for the research of glycocalyx-related diseases.

## Hyaluronic Acid Synthase

Unlike other glycosaminoglycans synthesized in the Golgi apparatus, hyaluronic acid is synthesized on the plasma membrane by one or more of the three hyaluronic acid synthetases (HAS 1–3) using UDP-glucuronic acid and UDP-n-acetylglucosamine as substrates (Itano et al., 1999; Mambetsariev et al., 2010; Torronen et al., 2014). Hyaluronic acid synthases 1 and 2 are responsible for the synthesis of high-molecular-weight hyaluronic acid. The properties of high- and low-molecular-weight morphology are very different: the former enhances the barrier function of endothelial cells; the latter destroys endothelial cells and induces toll-like receptors 2 and 4 (Zhang et al., 2018). In mammals, the deletion of the HAS 2 gene is fatal to mouse embryos (Moretto et al., 2015); however, mice lacking the HAS1 and/or HAS3 genes are normal and viable (Shakya et al., 2015). HAS2 can be modified by O-linked n-acetylglucosamine, thereby improving the stability and activity of HAS2. HAS2 and 3 synthesize hyaluronic acid from the hyaluronic acid shell; these two enzymes are isozymes (Itano et al., 1999).

Atherosclerosis and thrombosis, as large-vessel diseases of diabetes, are closely related to HAS, promoting the production of interstitial hyaluronic acid. Interstitial hyaluronic acid is synthesized under conditions that promote endoplasmic-reticulum stress. Endoplasmic-reticulum-stress-induced hyaluronic acid response leads to the formation of hyaluronic acid coats, thereby forming monocyte/macrophage adhesion (Sakr et al., 2008). PDGF-BB stimulates HAS2 expression, thereby enhancing PDGF-BB-induced migration through the formation of extracellular hyaluronic acid coats (Evanko et al., 1999). Thrombin stimulates HAS2 expression by activating the protease activation receptor (PAR) 1. When thrombi are incorporated into advanced complex lesions, thrombin is released, which may promote further plaque growth by stimulating hyaluronic acid synthesis (Evanko et al., 1999). In addition to indirect proinflammatory effects through the formation of foam cells, oxidized lipopolysaccharides are also a regulator of HAS2 and HAS3 expression in vascular smooth muscle cells, which may be a feed-forward mechanism to accelerate the formation of lesions (Viola et al., 2013). Unlike HAS3-mediated interstitial hyaluronic acid synthesis, the glycocalyx is also rich in hyaluronic acid, and can protect white blood cells and platelets from adhesion. The atherosclerotic effects of CD44 and toll-like receptors were demonstrated (Mullick et al., 2005; Bollyky et al., 2010). In multiple sclerosis, hyaluronic acid was shown to be important for T helper 1 cell polarization (Nagy et al., 2010). Hyaluronic acid is presented to dendritic cells as part of T-cell immune synapses. The absence of HAS3 reduces the polarization of T helper 1 cells. Has3/Apoe-deficient mice had reduced macrophage-driven inflammation and development of atherosclerotic lesions. Therefore, inhibition of hyaluronic acid synthesis can reduce atherosclerosis (Gupta et al., 1997; Buono et al., 2005; Fischer, 2019). In addition, a previous paper indicated that HAS3 knockout does not affect synovial-cell proliferation, but rather reduces synovial-cell migration (Nagy et al., 2010). In smooth muscle cells and T cells, HAS3 is induced by leukocyte-driven cytokines like IL-β and TNF-α. Inflammatory factors (TNF-α) first promote the mRNA expression of HAS2 and CD44, and then their protein expression (Vigetti et al., 2010). HAS3 gene knockdown inhibits the smooth muscle cell regulation of neointimal hyperplasia, atherosclerosis, T helper 1 cell polarization, and macrophage-driven inflammation. In turn, HAS3 promotes smooth muscle cell proliferation and migration, and provides a rich hyaluronic acid matrix, which is reshaped by immune cells through the CD44 signaling pathway and triggers deeper inflammation (Cuff et al., 2001). On the other hand, the synthesis of interstitial hyaluronic acid promotes the development of lesions by stimulating the migration and proliferation of smooth muscle cells, and locally and systemically produces important proinflammatory stimuli by promoting T helper 1 cell polarization (Bollyky et al., 2010). HAS3 also systemically stimulates T helper 1 cell polarization through interferon-γ, TNF-α, and interleukin 2 (IL2), thereby driving monocyte-/macrophage-mediated inflammation (Bollyky et al., 2009). These local and systemic proinflammatory effects are contrary to the protective effect of hyaluronic acid on endothelial glycogen against atherosclerosis (Buono et al., 2005).

Elevated vascular endothelial growth factor (VEGF) A levels are thought to cause glomerular endothelial-cell (GEnC) dysfunction and proteinuria in diabetic nephropathy (Onions et al., 2019) by regulating the synthesis of hyaluronic acid (Foster et al., 2013). Oltean et al. (2015) indicated that the induction of VEGF-A_165_b upregulation in mouse podocytes prevents functional and histological abnormalities in diabetic nephropathy. VEGF-A_165_b normalizes glomerular permeability through the phosphorylation of vascular endothelial growth factor receptor 2 in glomerular endothelial cells, and reverses the damage of glomerular endothelial glycogenases by diabetes. These results indicate that VEGF-A_165_b protects blood vessels and improves diabetic nephropathy through endothelial cells (Oltean et al., 2015). In addition, Onions et al. (2019) speculated that VEGF-C can offset these effects of VEGF-A, protect the glomerular filtration barrier, and reduce proteinuria. VEGF-C reduces the development of diabetic nephropathy, prevents the change of vascular endothelial growth factor receptors in diabetic glomeruli, and promotes glomerular protection and endothelial barrier function (Onions et al., 2019).

Another way in which hyaluronic acid synthesis responds to glucose metabolism is by the phosphorylation of AMP-activated protein kinase (AMPK), which inactivates HAS2. The concentration of hexosamine in cells determines HAS2 expression of controlling HAS2 transcription factors, such as the O-GlcNAcylation of YY1 and SP1. In addition, research found that inflammatory factors can promote the expression of NF-КB. Then, the mRNA of HAS2 and CD44 is upregulated, thereby promoting the expression of related proteins (Vigetti et al., 2010). Previous studies also found that, if hyaluronic acid synthesis is inhibited, Type 2 insulin resistance caused by adipose-tissue inflammation improves. Hyaluronic acid is related to autoimmunity. Hyaluronic acid fragments in body tissue can cause hyaluronidase degradation and nonenzymatic degradation in response to oxidative stress (Combadiere et al., 2008).

In short, when cells are in inflammation or other conditions, the glycocalyx degrades and produces hyaluronic acid fragments. These hyaluronic acid fragments, on the one hand, trigger the expression of inflammatory factors and form a vicious cycle of glycocalyx degradation. On the other hand, hyaluronic acid fragments promote HAS expression and form a hyaluronic acid coat on the glycocalyx surface. These interstitial hyaluronic acids are different from the hyaluronic acid present in the glycocalyx itself. They cause the adhesion of macrophages and monocytes, and then diseases such as atherosclerosis and thrombosis. Therefore, studying the relationship between hyaluronic acid synthase and glycocalyx degradation is of great significance for macrovascular-disease research.

## Conclusion and Prospection

**FIGURE 1:**
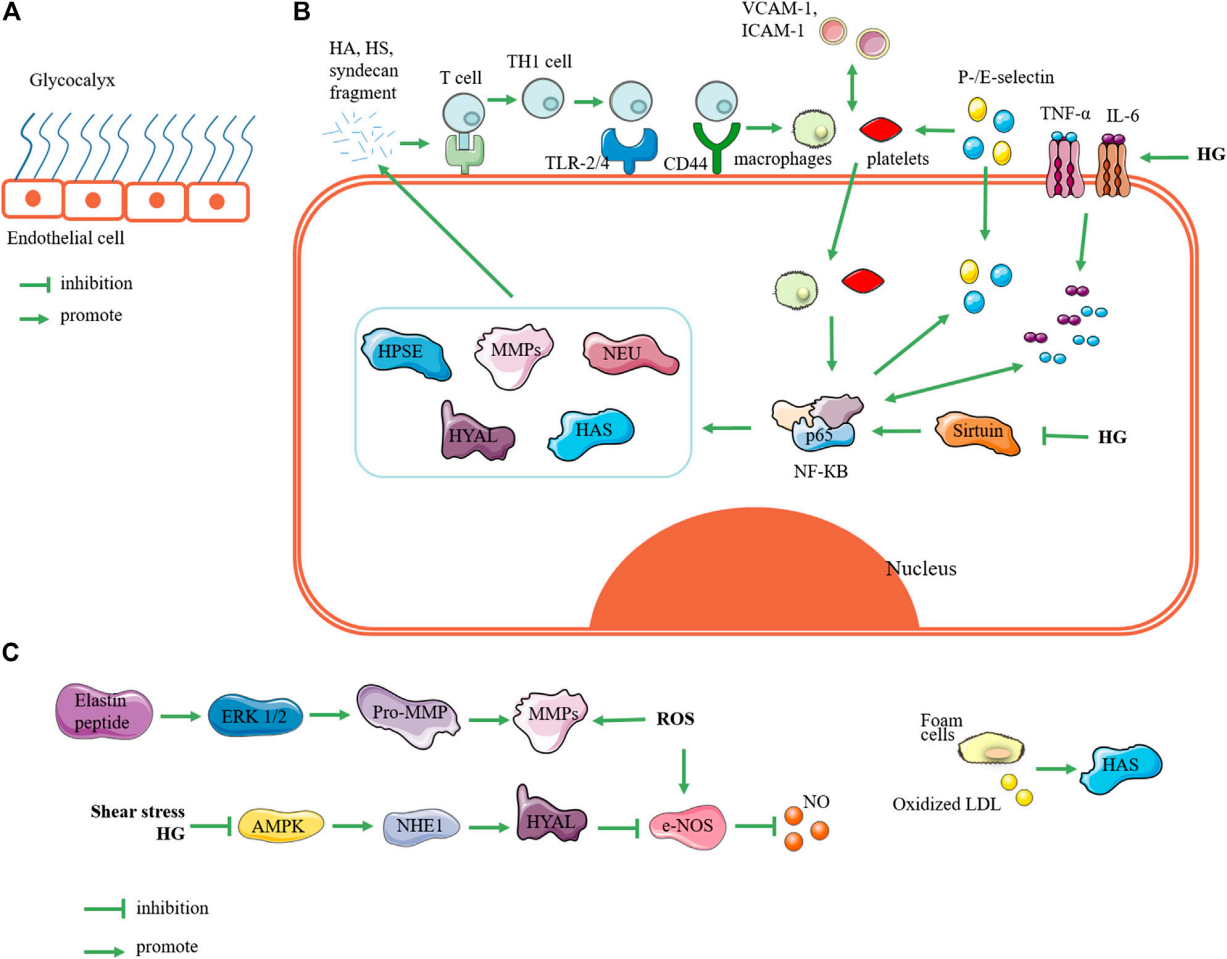
Related mechanisms of enzymes regulating glycocalyx. **(A)** State of glycocalyx under normal conditions. **(B)** Glycocalyxglycocalyx state under hyperglycemia. Elevated blood sugar triggers expression of inflammatory factors such as TNF and IL-6, which can trigger upregulation of NF-КB expression; in turn, this triggers upregulation of HPSE, MMPs, NEU, HYAL, and HAS. These enzymes degrade glycocalyx to produce fragments of hyaluronic acid, heparan sulfate, and syndecan. These fragments activate receptors such as toll-like receptors and CD44, so that T cells are presented and activated into T helper 1 cells. Furthermore, they induces monocyte adhesion such as macrophages and platelets. On the one hand, monocyte adhesion promotes expression of vascular cell adhesion molecule 1 and intercellular adhesion molecule 1, and it triggers the upregulation of P-/E-selectin expression; on the other hand, it can trigger the phosphorylation of NF-КB at position 65 and activate NF-КB. This forms a vicious circle of glycocalyx degradation. In addition, high glucose can inhibit the expression of sirtuin, which can upregulate the expression of NF-КB. **(C)** Relating other pathways for glycocalyx degradation, reactive oxygen species and elastin peptides can trigger upregulation of MMP expression; shear stress and hyperglycemia can upregulate Na^+^-H^+^ exchanger 1 through AMP-activated protein kinase (AMPK) pathway to upregulate HYAL expression; foam cells and oxidized low-density lipoprotein can promote HAS expression. Note: HPSE, heparanase; MMP, matrix metalloproteinase; NEU, neuraminidase; HYAL, hyaluronidase; HAS, hyaluronic acid synthase; HG: high glucose; HA: hyaluronic acid; HS: heparan sulfate; Th1 cell, T helper 1 cell; TLR-2/4, toll-like receptors 2 and 4; VCAM-1, vascular cell adhesion molecule-1; ICAM-1, intercellular adhesion molecule-1; ROS, reactive oxygen species; NHE1, Na^+^-H^+^ exchanger 1; LDL, low-density lipoprotein.

The glycocalyx can regulate the interactions between vascular endothelial cells and cytokines, platelets, and leukocytes (Vink et al., 2000; Mulivor and Lipowsky, 2002; Constantinescu et al., 2003; Florian et al., 2003). When the glycocalyx is damaged, it causes endothelial-cell inflammation, leukocyte rolling, platelet adhesion, thrombus and atherosclerosis in the aorta, and permeability changes in the microbes (Nieuwdorp et al., 2005). As early as 2004, Brownlee (2005) summarized three pathways of diabetes-induced complications: the polyol pathway, the advanced glycation end-product product glycosylation process, and the PKC pathway. Superoxide production by the mitochondrial electron transport chain was found to be a common pathway of the three pathways of glucose elevation and hyperglycemia damage (Nishikawa et al., 2000; Brownlee, 2005). After these pathways trigger the initial glycocalyx damage, the heparan sulfate, hyaluronic acid, and syndecan fragments produced by glycocalyx degradation promote further inflammation. We summarize the relevant mechanism in Figure 1. We speculate that the inflammation caused by high glucose is the main cause of glycocalyx destruction. The expression of inflammatory factors such as NF-КB, TNF-α, and IL-6 promotes the upregulation of related enzymes at the RNA and/or protein levels. The upregulation of the enzyme triggers glycocalyx degradation, producing heparan sulfate and syndecan fragments. First, fragments produced by glycocalyx degradation induce the polarization of T helper 1 cells, and subsequently induce the upregulation of CD44 and toll-like receptor 2 and 4 expression (Zeng, 2017). On the one hand, this causes the adhesion and rolling of macrophages and monocytes; on the other hand, it activates the NF-КB pathway and upregulates the expression of HPSE, MMPs, HYAL, HAS, and NEU (Sieve et al., 2018a). Then, the hyperglycemic inflammatory environment and the degradation of the glycocalyx form a vicious circle (Lemkes et al., 2010; Dogne et al., 2016; Szot et al., 2019). In this cycle, glycocalyx-degradation-related enzymes play important roles. In addition, oxidative stress, the abnormal expression of NO, and shear stress affect the expression of glycocalyx-degrading enzymes, which in turn trigger changes in the glycocalyx. In many related diseases, the expression of these enzymes is upregulated. Therefore, inhibiting the expression of enzymes at the protein or RNA level is a new way to treat vascular complications. However, hyaluronidase deletion can reduce the production of HA fragments and glycocalyx damage (Dogne et al., 2016). In humans and mice, the complete absence of hyaluronidase 1 can cause mucopolysaccharidosis IX (Natowicz et al., 1996; Martin et al., 2008). In mammals, the deletion of the HAS 2 gene is fatal to mouse embryos (Moretto et al., 2015). Therefore, although enzyme inhibitors are a good direction for drug development, it is still necessary to pay attention to the side effects of excessive enzyme inhibition. Nevertheless, it is of practical significance to use these enzymes as therapeutic targets to seek treatment for diabetic vascular complications. Furthermore, the current research on glycocalyx degradation related enzymes is focused on hyaluronidase, hyaluronic acid synthase, and heparan sulfate. While neuraminic acid and chondroitin sulfate are part of the glycocalyx, we speculate that these fragments produced by glycocalyx degradation will also have a huge impact. Therefore, exploring the role of chondroitin sulfate and neuraminidase in glycocalyx degradation is a direction worth exploring.

## Author Contributions

NW and QZ gave the first idea for the manuscript. ZL wrote the manuscript, and NW, JW and QZ revised the manuscript. All authors listed have made substantial, direct, and intellectual contribution to the work and approved it for publication.

## Funding

This work is supported by the Science and Technology Service Network Initiative (KFJ-STS-QYZD-195), Shandong Province major science and technology innovation project (2019JZZY010818) and Special Open Fund of Laboratory for Marine Biology and Biotechnology, Qingdao Pilot National Laboratory for Marine Science and Technology (No. OF2020NO02).

## Conflict of Interest

The authors declare that the research was conducted in the absence of any commercial or financial relationships that could be construed as a potential conflict of interest.
